# Extracellular Matrix Proteins Confer Cell Adhesion-Mediated Drug Resistance Through Integrin α_*v*_ in Glioblastoma Cells

**DOI:** 10.3389/fcell.2021.616580

**Published:** 2021-03-23

**Authors:** Qi Yu, Weikun Xiao, Songping Sun, Alireza Sohrabi, Jesse Liang, Stephanie K. Seidlits

**Affiliations:** ^1^Department of Bioengineering, University of California, Los Angeles, Los Angeles, CA, United States; ^2^Department of Neurosurgery, Shengjing Hospital of China Medical University, Shenyang, China; ^3^Jonsson Comprehensive Cancer Center, University of California, Los Angeles, Los Angeles, CA, United States; ^4^Brain Research Institute, University of California, Los Angeles, Los Angeles, CA, United States; ^5^Broad Stem Cell Research Center, University of California, Los Angeles, Los Angeles, CA, United States

**Keywords:** cell adhesion-mediated drug resistance, integrin α_*v*_, glioblastoma, extracellular matrix, laminin, vitronectin, fibronectin

## Abstract

Chemotherapy resistance to glioblastoma (GBM) remains an obstacle that is difficult to overcome, leading to poor prognosis of GBM patients. Many previous studies have focused on resistance mechanisms intrinsic to cancer cells; the microenvironment surrounding tumor cells has been found more recently to have significant impacts on the response to chemotherapeutic agents. Extracellular matrix (ECM) proteins may confer cell adhesion-mediated drug resistance (CAMDR). Here, expression of the ECM proteins laminin, vitronectin, and fibronectin was assessed in clinical GBM tumors using immunohistochemistry. Then, patient-derived GBM cells grown in monolayers on precoated laminin, vitronectin, or fibronectin substrates were treated with cilengitide, an integrin inhibitor, and/or carmustine, an alkylating chemotherapy. Cell adhesion and viability were quantified. Transcription factor (TF) activities were assessed over time using a bioluminescent assay in which GBM cells were transduced with lentiviruses containing consensus binding sites for specific TFs linked to expression a firefly luciferase reporter. Apoptosis, mediated by p53, was analyzed by Western blotting and immunocytofluorescence. Integrin α_*v*_ activation of the FAK/paxillin/AKT signaling pathway and effects on expression of the proliferative marker Ki67 were investigated. To assess effects of integrin α_*v*_ activation of AKT and ERK pathways, which are typically deregulated in GBM, and expression of epidermal growth factor receptor (EGFR), which is amplified and/or mutated in many GBM tumors, shRNA knockdown was used. Laminin, vitronectin, and fibronectin were abundant in clinical GBM tumors and promoted CAMDR in GBM cells cultured on precoated substrates. Cilengitide treatment induced cell detachment, which was most pronounced for cells cultured on vitronectin. Cilengitide treatment increased cytotoxicity of carmustine, reversing CAMDR. ECM adhesion increased activity of NFκB and decreased that of p53, leading to suppression of p53-mediated apoptosis and upregulation of multidrug resistance gene 1 (MDR1; also known as ABCB1 or P-glycoprotein). Expression of Ki67 was correlative with activation of the integrin α_*v*_-mediated FAK/paxillin/AKT signaling pathway. EGFR expression increased with integrin α_*v*_ knockdown GBM cells and may represent a compensatory survival mechanism. These results indicate that ECM proteins confer CAMDR through integrin α_*v*_ in GBM cells.

## Introduction

Glioblastoma (GBM) is the most common primary brain tumor and the most aggressive in nature. For many decades, the standard therapy for GBM remains the same, including maximum feasible surgical resection, followed by radiation (XRT) plus concomitant chemotherapy with temozolomide (TMZ), and then followed by adjuvant TMZ (Ellor et al., 2014; Patel et al., 2014). The combined therapy resulted in an improved median overall survival from 12.1 to 14.6 months and an increase in the 2 years survival rate from 10 to 27% (Miranda et al., 2017). The Gliadel wafer (Arbor Pharmaceuticals, Atlanta, GA) is a carmustine-impregnated wafer that is placed in the surgical cavity after maximal tumor resection. Prospective data regarding use of carmustine wafers included all high-grade gliomas, and some of the survival benefits were largely a result of grade 3 patients with long-term survival (Chowdhary et al., 2015). Some retrospective studies also suggest a modest gain of 2–3 months for patients with GBM (Dixit et al., 2011). Nevertheless, the rising chemotherapeutic agent resistance leading to the treatment failure is still a challenge and has been one of the priorities in neuroscience. Additional concomitant chemotherapeutics for newly diagnosed GBM have not shown an incremental survival benefit. Concomitant bevacizumab with TMZ against newly diagnosed GBM in two large phase III trials showed prolonged progression-free survival but failed to show survival benefit (Chinot et al., 2014; Gilbert et al., 2014). Similarly, cilengitide, as an selective α_*v*_β_3_ and α_*v*_β_5_ integrin inhibitor, als although cilengitide exhibited antitumor activity in phase II trials. it failed to show overall survival improvement in patients with methylated MGMT promoter in phase III trials (Stupp et al., 2014; Nabors et al., 2015). Rindopepimut is a peptide vaccine against the most common epidermal growth factor receptor (EGFR) deletion mutation (EGFR variant III) in GBM. Although single-arm phase II studies showed promising results, a randomized phase III trail (ACT IV) failed to show benefit over the control group (Weller et al., 2017).

Advanced knowledge has been established with regard to the mechanism of chemoresistance in GBM. One reason why GBM has a very poor prognosis is related with the lack of successful drug delivery across the physiologic barriers, especially the blood–brain barrier (BBB), which protects the central nervous system (CNS) from the passage of foreign and harmful substances through the bloodstream (Pourgholi et al., 2016). The relevant mechanisms underlying the barrier function include enzymatic barrier, transport barrier (para-cellular and transcellular), immunologic barrier, and efflux transport systems (Hendricks et al., 2015). Mutation of DNA repair systems is another cause responsible for the chemoresistance such as enhanced MGMT activity, impaired DNA mismatch repair system, and enhanced base excision repair system (Thon et al., 2013; Messaoudi et al., 2015; Motegi et al., 2019). In addition, other factors have been shown to interfere with TMZ activity, contributing to the poor prognosis of GBM. Such factors include EGFR, PI3K/AKT/mTOR pathway (the mechanistic target of rapamycin), galectin-1, p53, murine double minute 2 (Mdm2), ATP-binding cassette transports, phosphatase and tensin homolog (PTEN), isocitrate dehydrogenase (IDH-1), and cell cycle checkpoint pathways (Messaoudi et al., 2015; Tivnan et al., 2015; Pourgholi et al., 2016; Yan et al., 2016; Kim and Kim, 2020; Pine et al., 2020). More recently, a series of publications identified cell adhesion to the extracellular matrix (ECM) as a key determinant among the myriad of microenvironmental factors impacting cancer cell resistance (Xiao et al., 2017). It has been reported that adhesion to laminin, vitronectin, or fibronectin confers cell adhesion-mediated drug resistance (CAMDR) in various cancer models (Fei et al., 2014; Nakagawa et al., 2014; Sun et al., 2014; Sanchez et al., 2019). Data have revealed that α_*v*_β_3_ and α_*v*_β_5_ integrins mediate the interaction between endothelial cells and components of the ECM (Varner et al., 1995). Integrin α_*v*_β_3_ binds to Arg-Gly-Asp (RGD) in vitronectin, fibronectin, and fibrinogen, among other substrates. Cilengitide, one of the cyclic RGD peptides, was found to disrupt VE-cadherin localization at cell junctions, increase endothelia monolayer permeability, and restrain angiogenesis *in vitro* and *in vivo* (Chowdhary et al., 2015).

The p53 transcription factor (TF) was initially known as the guardian of the genome due to the fact that it prevents the proliferation of cells with damaged nuclear DNA (Olotu and Soliman, 2019; Horikawa, 2020). However, p53 acts as a TF to regulate the expression of a variety of genes that coordinate the DNA damage responses. On the one hand, it can initiate apoptosis through death receptor and mitochondrial pathway. On the other hand, it can arrest growth by holding the cell cycle at the G1/S regulation point through p53-dependent p21^*WAF*1/CIP1^, which binds to the G1-S/CDK complex and inhibits their activity.

Carmustine [1,3-bis(2-chloroethyl)-1-nitrosourea (BCNU)] was first introduced for chemotherapy against malignant gliomas in the 1980’s (Walker et al., 1978; Walker et al., 1980). During the past 30 years, the evaluation of the role of chemotherapy has not produced impressive results to date. Beyond TMZ, the US Food and Drug Administration (FDA) has approved two other agents for treatment of newly diagnosed malignant glioma till now: carmustine wafers and bevacizumab (Affronti et al., 2009). Gliadel wafers (Arbor Pharmaceuticals, Atlanta, GA) are commercial products of biodegradable copolymers (prolifeprospan 20) impregnated with carmustine. Wafer efficacy has been well documented (Stewart, 2002). Subsequent trials revealed increased benefit from combining Gliadel wafers with XRT/TMZ. Median overall survival tended to be improved by 3–4 months beyond that observed for Gliadel wafers or TMZ when used alone in the respective III trials (Ashby et al., 2016).

In this study, we found that laminin, vitronectin, and fibronectin, three main components of ECM proteins, could affect CAMDR in GBM cells. Enrichment of these proteins in the microenvironment promotes tumor cell proliferation through integrin α_*v*_-mediated FAK/paxillin/AKT signaling pathway and suppresses p53-mediated apoptosis. In addition, we found that the efflux transporter ABCB1 was elevated with ECM adhesion. Compensatory activation of EGFR occurred when integrin α_*v*_ was knocked down. These findings will provide promising insights to overcome chemotherapeutic resistance for GBM.

## Materials and Methods

### Cell Lines, Cell Culture, and Treatment Setup

The GBM cell line U87MG was obtained from American Type Culture Collection (ATCC) and cultured in Dulbecco’s modified Eagle’s medium (DMEM) supplemented with 10% fetal bovine serum (FBS) and 1% penicillin/streptomycin. Primary GBM cell line HK308 (patient background: a 50-year-old female with recurrent GBM with wild-type IDH 1/2 received XRT, TMZ, and Avastin treatment) was established from patient tumors in accordance with UCLA Institutional Review Board protocol 10-000655 and generously gifted by Dr. Harley Kornblum at UCLA. Authentication was conducted by immunoblot studies (Laks et al., 2016). Primary GBM6 (patient background: a 65 years old male with newly diagnosed GBM with mutant p53 and amplification of EGFR vIII mutant received XRT and TMZ treatment) was obtained from Dr. David Nathanson and authenticated by DNA fingerprinting (Sarkaria et al., 2006; Akhavan et al., 2013). DMEM/F12 with G21 (Gemini Bio, Sacramento, CA, United States, 1:50), 50 ng/ml of EGF (PeproTech, Rocky Hill, NJ, United States), 20 ng/ml of FGF-2 (PeproTech), 25 μg/ml of heparin (Sigma-Aldrich, St. Louis, MO, United States), and 1% penicillin/streptomycin was used for primary GBM cell line culture. All cell lines were used fewer than 30 passages and incubated in a 5% CO_2_-humidified incubator at 37°C.

ECM proteins including laminin (Thermo Fisher Scientific, Waltham, MA, United States), vitronectin and fibronectin (Sigma-Aldrich, St. Louis, MO, United States) were used, respectively, as coating protein in a 12-well plate at a dilution of 10 μg/ml. The plate was then incubated at 37°C for over 4 h before the cells were seeded. Carmustine was purchased from Sigma-Aldrich and dissolved at 100 mmol/L in 100% ethanol for stocking. It was then added in culture medium at a final concentration of 100 μmol/L. Ethanol alone was used as the negative control. Cilengitide (Sigma-Aldrich, St. Louis, MO, United States) was dissolved in phosphate-buffered saline (PBS) as 10 mmol/L of stock and then added in culture medium at a final concentration of 50 μmol/L.

### Tissue Microarray

Tissue microarray (TMA) containing 34 GBM samples and 19 other low-grade CNS tumors were employed to analyze laminin, vitronectin, and fibronectin expressions. All the patients had given informed consent, and collection of these tissue samples had been studied for other researches before (Choe et al., 2003; Guo et al., 2009; Lu et al., 2009). TMA was a high-throughput screening platform that enables a bunch of patient tumor samples to be analyzed on the same slide. It was constructed using a 0.6 mm needle to exact 54 representative tumor tissue cores from the paraffin-embedded tissue blocks (Akhavan et al., 2013). Theses cores were placed in a grid pattern into two recipient paraffin blocks, from which tissue sections were cut for immunohistochemistry (IHC) analysis, as previously described (Zhou et al., 2015). Briefly, the slides were first deparaffinized, followed by blocking with 30% normal donkey serum for 10 min. Then the sections were incubated with the primary laminin, vitronectin, and fibronectin antibodies (Thermo Fisher, PA1-16730/PA5-27909/PA1-26205, respectively; Waltham, MA, United States, dilution 1:500) overnight at 4°C. Appropriate secondary antibodies were applied for 30 min. Negative controls were carried out by replacement of the primary antibody with substituting PBS. Images were scored and calculated by Fisher’s exact test.

### Cell Adhesion Assay

A 96-well plate was coated with PBS (as negative control) and 10 μg/ml of laminin, vitronectin, and fibronectin, over 4 h at 37°C. To block any remaining protein binding sites on the plate, coating solutions were removed, and then 1% bovine serum albumin (BSA) was added for another hour. Appropriate density of GBM cell suspension (10,000 cells/well for GBM6; 5,000 cells/well for U87MG and HK308) was seeded, followed by another 2 h incubation. Non-adherent GBM cells were removed by careful washing two times with PBS. Then, 100% ethanol was used to fix the adherent cells for 15 min followed by 0.1% crystal violet (Thermo Fisher Scientific, Pittsburgh, PA, United States, dissolved in 100% ethanol) staining for another 30 min. After excessive stain was removed and 0.3% Triton-X was applied to lyse cells, the absorbance was measured at 570 nm using a microplate reader (BioTek, Winooski, VT, United States). The percentage of adhesion was determined by dividing the corrected (background subtracted) optical density of adherent cells by the total corrected optical of cells added to each microplate well and multiplying by 100%. Experiments were repeated three times with five replications per experiment.

### Cell Viability Assay

Cell viability and drug–response curves were assessed using a CellTiter 96 AQ_*ueous*_ One Solution (MTS) kit (Promega, Madison, WI, United States) as described before (Kong et al., 2015). Briefly, GBM cells (5,000 cells/well for GBM6; 3,000 cells/well for U87MG and HK308) were cultured on a 96-well plate precoated with PBS, laminin, vitronectin, or fibronectin for 24 h. Cells were then treated with 100 μM of carmustine or ethanol (as control). At the end of the treatment, 20 μl/well of MTS solution was added and incubated at 37°C for 2 h. Absorbance was measured at 490 nm. All data points were set up with five replicates for each experiment. The IC_50_ was determined by GraphPad Prism Software Version 7 (San Diego, CA, United States).

### Immunofluorescent Staining

GBM cells were fixed with 4% paraformaldehyde for 20 min at room temperature and permeabilized with a blocking solution containing 5% BSA and 0.01% Triton X-100 diluted in PBS. Then, cells were incubated overnight at 4°C with anti-Ki67 primary antibody (1:200, Thermo Fisher Scientific, Waltham, MA, United States). Goat anti-rabbit secondary antibody was then added at a dilution 1:500 for 2 h. Secondary antibody alone without primary antibody was used as negative control. All the GBM cells were later counterstained with Hoechst 33242 (Thermo Fisher Scientific, Waltham, MA, United States). Glass coverslips were mounted with fluorescence Mounting Medium (SouthernBiotech, Birmingham, AL, United States). Images were captured with an AXIO-Observer inverted microscope equipped for wide-field fluorescence and phase contrast (Zeiss, Oberkochen, Germany).

### Lentiviral Construction and Transfection

Plasmids pTA-/p53-/NFκB-/c-myc-FLuc were kindly obtained from Dr. Lonnie D. Shea at Northwestern University. The plasmids were designed so that each contained a consensus binding sequence for a particular TF in the enhancer region upstream of a minimal TATA-box promoter driving expression of the reporter gene firefly luciferase (Supplementary Table 1; Penalver Bernabe et al., 2016). TP53bp1 was a gift from Nicola Burgess-Brown (Addgene plasmid #73252). ITGAV shRNA was obtained from Dharmacon (V2LHS_133468, Lafayette, CO, United States). Lentivirus was produced by co-transfecting HEK-293T cells with a third-generation packaging system (Dull et al., 1998). As quantified by lentix-rtPCR kit (Takara), transduction was performed with a virus concentration of 2,000 physical particles/cell. Fresh media were replaced 24 h after transfection. TA-/p53-/NFκB-/c-myc-FLuc, integrin α_*v*_ knockdown, and TP53bp1^+^ stable cell lines were created and continuously cultured for 3 days before use in the subsequent assay. All experiments with regard to the virus were performed on BSL2 laboratory under relevant management regulations.

### Bioluminescence Assay

TF activity was assessed by bioluminescence imaging of firefly luciferase using an IVIS imaging system (Caliper Life Sciences, Hopkinton, MA, United States) as described previously (Bellis et al., 2011). D-Luciferin (Sigma-Aldrich, St. Louis, MO, United States) was added as the substrate for Fluc at 1 μM/well, followed by incubation for 1 h. Exposure time was 5 min, and images were taken every 24 h for 3 days. After each time point of any dynamic imaging experiment, the medium was refreshed. Normalized TF activity was determined by dividing the normalized light emission for target TFs by the average normalized light emission for TA. Each condition was performed in triplicate.

### Western Blotting Antibody Information Table

Proteins were extracted by lysing GBM cell lines in radioimmunoprecipitation assay (RIPA) buffer with a protease/phosphatase inhibitor cocktail. Then the sodium dodecyl sulfate–polyacrylamide gel electrophoresis and transfer were performed, followed by blocking in 5% BSA as described previously (Xiao et al., 2018; Yu et al., 2018). Relevant primary antibodies were used for detecting target bands overnight at 4°C (integrin α_*v*_/β_1_/β_3_/β_5_, t-/p-FAK, p-paxillin, t-/p-AKT, t-/p-EGFR, p53, cleaved PARP, ABCB1, p-ERK1/2, and cyclin D1, obtained from Cell Signaling Technology, Danvers, MA, United States, dilution 1:1,000). Horseradish peroxidase (HRP) goat anti-mouse IgG or anti-rabbit IgG were used as secondary antibodies (dilution 1:2,000). Immunoreactive bands were visualized using Clarity ECL substrate (Bio-Rad, Hercules, CA, United States) and imaged (MyECL imager) without overexposing the target bands. Equal loading was assessed after probing the same membrane with anti-GAPDH antibody (Thermo Fisher Scientific, Waltham, MA, United States). Images of blots were analyzed using ImageJ (NIH).

### Statistical Analysis

Statistical analysis was performed using GraphPad Prism Software Version 7 (San Diego, CA, United States). Two-way ANOVA test was applied to examine the statistical significance followed by Dunnett’s test as a *post hoc* test for within- or between-group comparisons. Probability values less than 0.05 were regarded as statistically significant.

## Results

### Extracellular Matrix Proteins Confer Cell Adhesion-Mediated Drug Resistance in Glioblastoma Cell Lines

**FIGURE 1:**
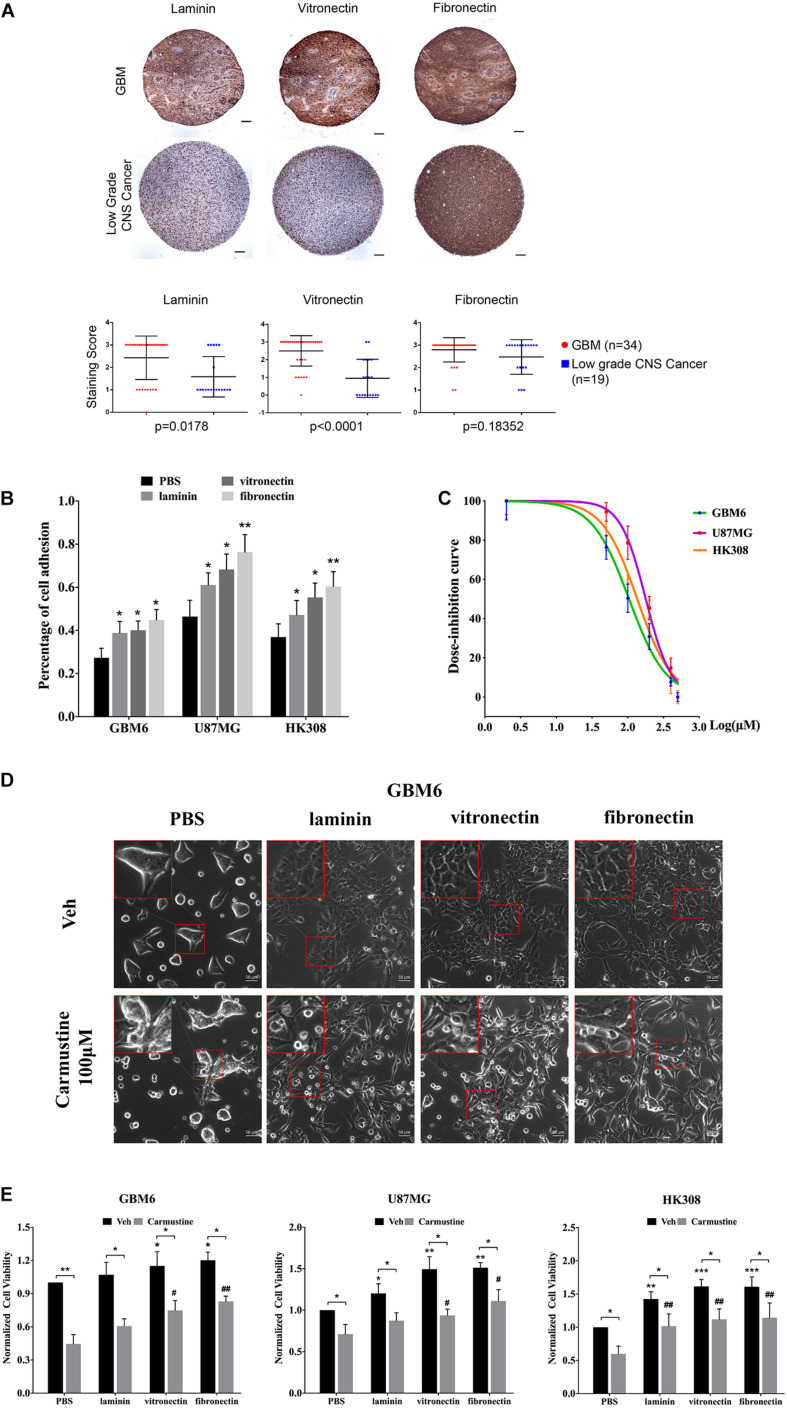
Extracellular matrix (ECM) proteins confer cell adhesion-mediated drug resistance (CAMDR) in glioblastoma (GBM) cell lines. **(A)** Tissue microarray (TMA) of expressions of laminin, vitronectin, and fibronectin in patient-derived GBM and low-grade central nervous system (CNS) tumors. Scale bars, 100 μm. GBM group, *n* = 34; low-grade CNS cancer, *n* = 19. **(B)** Attachment assay of GBM cell lines GBM6, U87MG, and HK308 on fibronectin, laminin, and vitronectin. Error bars, *SD* (*n* = 3). **(C)** Dose inhibition curve of carmustine on GBM6, U87MG, and HK308. Error bars, *SD* (*n* = 3). **(D)** Representative light images of GBM6 with or without carmustine [100 μM, phosphate-buffered saline (PBS) as control] treatment on precoated fibronectin, laminin, and vitronectin. Scale bars, 50 μm. **(E)** Cell viability assay of carmustine on GBM6, U87MG, and HK308. Error bars, *SD* (*n* = 3). *p < 0.05, **p < 0.01, ***p < 0.001, ^#^*p* < 0.05, ^##^*p* < 0.01.

To investigate the impact of ECM on GBM tumor cells, IHC of patient TMAs was used to analyze expression of laminin, vitronectin, and fibronectin in GBM and low-grade CNS tumors (Figure 1A). Compared with tumors in the low-grade CNS group, GBM tumors showed higher expression of laminin (*p* = 0.0178) and vitronectin (*p* < 0.0001). Fibronectin was widely expressed in both GBM and low-grade CNS tumor groups. An *in vitro* assay showed that fibronectin induced the highest degree of attachment for the U87 GBM cell line and two patient-derived GBM cell lines (GBM6 and HK308), followed by vitronectin and laminin, respectively (Figure 1B). All ECM proteins investigated significantly increased attachment over non-coated substrates (laminin, *p* < 0.05 for all; vitronectin, *p* < 0.05 for all; fibronectin, *p* < 0.05 for GBM6; *p* < 0.01 for U87MG and HK308).

Adhered cells were treated with carmustine, an alkylating agent that forms interstrand cross-links in DNA to prevent its replication and transcription. GBM cells were inhibited by carmustine in a dose-dependent manner, with a half maximal inhibitory concentration (IC_50_) of 106 ± 13 μM for GBM6, 179 ± 29 μM for U87MG, and 137 ± 27 μM for HK308 (Figure 1C). To assess whether ECM proteins conferred CAMDR, GBM cells were cultured on substrates precoated with PBS (as a negative control), laminin, vitronectin, or fibronectin for 24 h prior to treatment with carmustine (or vehicle control) for 48 h. In the absence of precoated ECM, carmustine treatment significantly reduced numbers of adhered cells (Figure 1D and Supplementary Figure S1). While some rounded cells were observed on ECM-coated substrates, more rounded cells were apparent on non-coated substrates after treatment. Quantitative assays showed a significant increase in cell viability when cultured on either vitronectin- or fibronectin-coated (compared with uncoated) substrates (vitronectin, *p* < 0.05 for GBM6, *p* < 0.01 for U87MG, *p* < 0.001 for HK308; fibronectin, *p* < 0.05 for GBM6, *p* < 0.01 for U87MG, *p* < 0.001 for HK308), while adhesion to laminin significantly increased viability of U87MG and HK308 (*p* < 0.05 for U87MG; *p* < 0.01 for HK308), but not GBM6, cells (Figure 1E). Treatment with carmustine for 48 h significantly decreased cell viability in all cases; however, ECM adhesion provided significant protection over non-coated substrates (Figure 1E, PBS, *p* < 0.05 for all cell lines; laminin, *p* < 0.05 for GBM6 and U87MG, *p* < 0.01 for HK308; vitronectin, *p* < 0.05 for U87MG, *p* < 0.01 for GBM6 and HK308; fibronectin, *p* < 0.01 for all).

### Cilengitide Reverses Cell Adhesion-Mediated Drug Resistance Through Integrin α_*v*_ in a Matrix-Specific Manner

**FIGURE 2:**
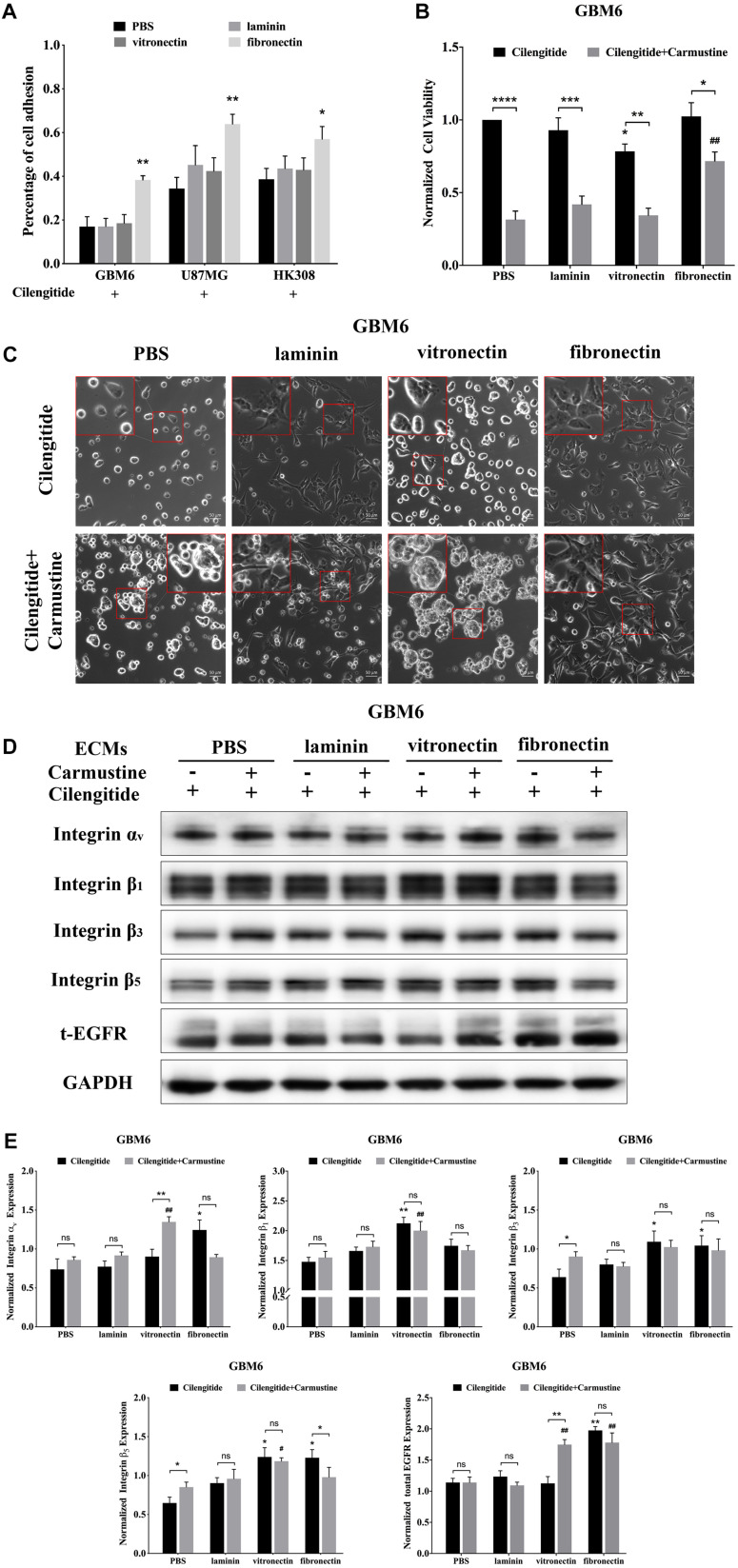
Cilengitide reverses cell adhesion-mediated drug resistance (CAMDR) through of integrin α_*v*_ in a matrix-specific manner. **(A)** Attachment assay of cilengitide treated glioblastoma (GBM) cell lines GBM6, U87MG, and HK308 on fibronectin, laminin, and vitronectin. Error bars, SD (*n* = 3). **(B)** Cell viability assay of carmustine and cilengitide on GBM6 cells. Error bars, *SD* (*n* = 3, *p < 0.05, **p < 0.01, ***p < 0.001, ****p < 0.0001, ^#^*p* < 0.05, ^##^*p* < 0.01). **(C)** Representative light images of GBM6 cells with or without carmustine and cilengitide [100 μM, phosphate-buffered saline (PBS) as control] treatment on precoated fibronectin, laminin, and vitronectin. Scale bars, 50 μm. **(D,E)** Representative western blot of GBM6 with or without carmustine and cilengitide (100 μM, PBS as control) treatment on integrin subunits (α_*v*_, β_1_, β_3_, and β_5_) and epidermal growth factor receptor (EGFR) expression (*n* = 3).

Cilengitide, a selective inhibitor of the integrin α_*v*_ receptor, decreases adhesion of GBM6, U87MG, and HK308 cells on precoated laminin and vitronectin, but not fibronectin (Figure 2A).

Compared with cells grown on substrates without precoated ECM protein, only coating with fibronectin results in higher levels of GBM6 cell viability (Figure 2B, *p* < 0.01). However, treatment with cilengitide only reduced viability of cells when cultured on vitronectin-coated substrates (Figure 2B, *p* < 0.05). Similarly, coating with fibronectin, but not laminin or vitronectin, leads to restoration of U87MG cell viability when treated with cilengitide plus carmustine (Supplementary Figure S2A, *p* < 0.05). U87MG shows less viability, with cilengitide on precoated laminin and vitronectin substrates (Supplementary Figure S2A, *p* < 0.05). On the contrary, cilengitide does not reverse cell viability of HK308 on any precoated substrates (Supplementary Figure S2A). In general, a loss of cell adhesion and spreading after cilengitide treatment (Figure 2C and Supplementary Figure S2B) was associated with increased efficacy carmustine treatment (Figure 2B and Supplementary Figure S2A). Cell responses after detachment by cilengitide are also different between cell types. For GBM6, detachment was only observed on precoated vitronectin, which resulted in higher chemotherapeutic toxicity. For U87MG, detachment occurred in all substrates except fibronectin, which leads to lower cell viability on relative substrates. Cilengitide did not affect cell adhesion and carmustine efficacy in HK308. Next, we investigated candidate integrins, which were required as the receptor for GBM6 cells responding to cilengitide administration on varying ECMs (Figures 2D,E). All integrin subunits evaluated were upregulated by cells cultured on fibronectin, when compared with those cultured on laminin or vitronectin (Figures 2D,E). Moreover, culture fibronectin-coated substrates also induced upregulation of EGFR expression.

Compared with GBM6, cells grown on uncoated substrates, cilengitide treatment induced upregulation of integrin subunits in an ECM-dependent way. Specifically, integrin α_*v*_ expression was higher in cells grown on fibronectin, integrin β_1_ in cells grown on vitronectin, and integrins β_3_ and β_5_ with cells grown on fibronectin or vitronectin (Figure 2E, *p* < 0.05). When treated with a combination of cilengitide and carmustine, shifts in integrin expression were again dependent on the type of ECM coating. Cells grown on uncoated substrates increased expression of integrins β_3_ and β_5_, while those on fibronectin downregulated integrins α_*v*_ and β_5_. When cultured on vitronectin, dual treatment with cilengitide and carmustine increased integrin α_*v*_ and total EGFR expression.

### Extracellular Matrix Induces Activation of NFκB and Deactivation of p53

**FIGURE 3:**
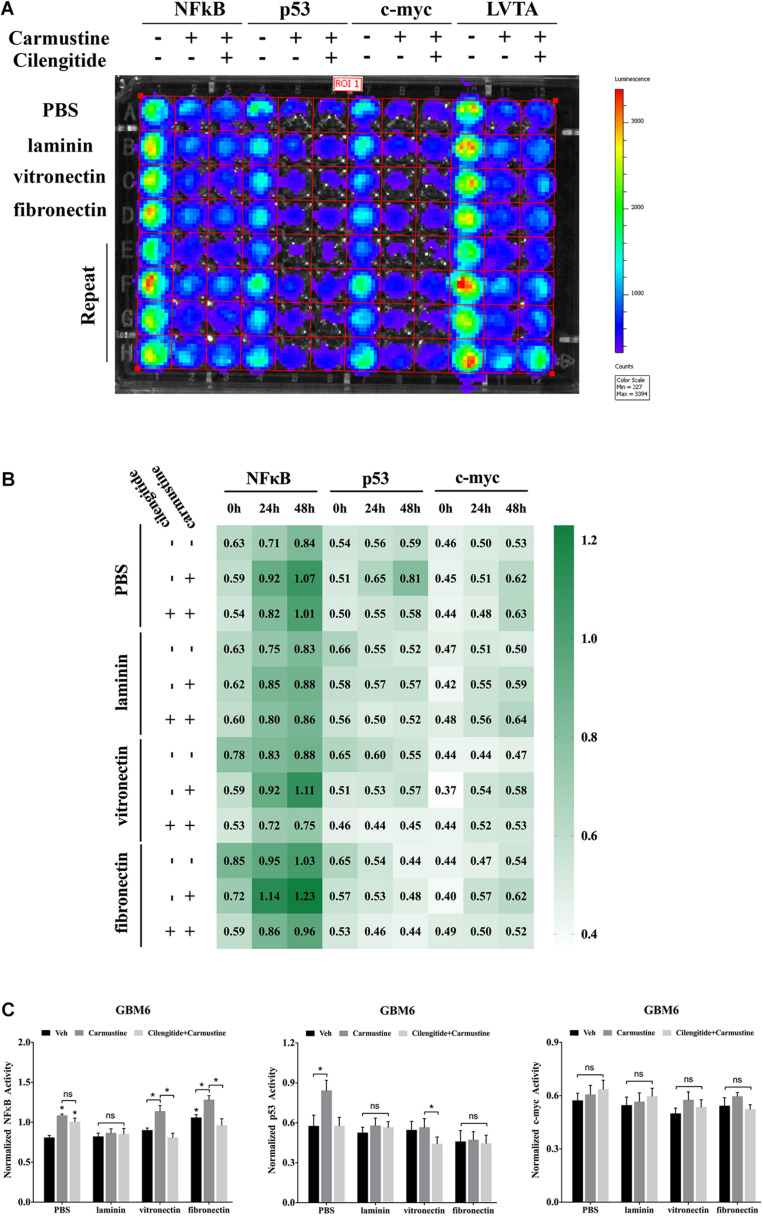
Extracellular matrix induces activation of NFκB and deactivation of p53. **(A)** Bioluminescence assay of GBM6 cells with or without carmustine and cilengitide on precoated extracellular matrix (ECM) proteins (*n* = 3). **(B)** Heatmap of sequential expression of transcription factors (TFs) in GBM6 cells over time with or without carmustine and cilengitide (*n* = 3). **(C)** Normalized TFs activity in GBM6 cells after 48 h treatment with or without carmustine and cilengitide on precoated ECM proteins (*n* = 3). *p < 0.05.

Binding events to consensus sequences for the TFs NFκB, p53, and c-myc in response to treatments were assessed using GBM6 cells transduced with bioluminescent reporters of TF activity when cultured on ECM-coated substrates (Figure 3A) as previously reported (Penalver Bernabe et al., 2016). In all cases, there was a trend towards increased NFκB reporter during the 48 h after seeding (Figure 3B). This trend was more pronounced with carmustine treatment. Activity of the p53 reporter was only affected in cells seeded on uncoated substrates and treated with both carmustine and cilengitide. There were no obvious effects of any condition on c-myc activity.

After 48 h of treatment with carmustine or carmustine and cilengitide, GBM6 cells on uncoated substrates exhibited significantly higher NFκB reporter activity (Figure 3C, *p* < 0.05). Activity of the p53 reporter increased in cells on uncoated substrates with carmustine treatment, but this effect was lost with combined treatment with carmustine and cilengitide. When seeded on vitronectin or fibronectin, NFκB activity increased significantly with carmustine treatment alone, but this effect was lost when treated with both carmustine and cilengitide (Figure 3C, *p* < 0.05). Activity of the p53 reporter significantly decreased with affected by dual treatment with carmustine and cilengitide in cells seeded on vitronectin (Figure 3C, *p* < 0.05). No other differences in p53 reporter activity were observed. Likewise, no significant changes in activities of NFκB, p53, and c-myc reporters were observed with laminin coating. No significant differences in c-myc reporter activities were observed in any conditions.

### Extracellular Matrix Suppresses p53-Mediated Apoptosis and Increases ABCB1 Expression

**FIGURE 4:**
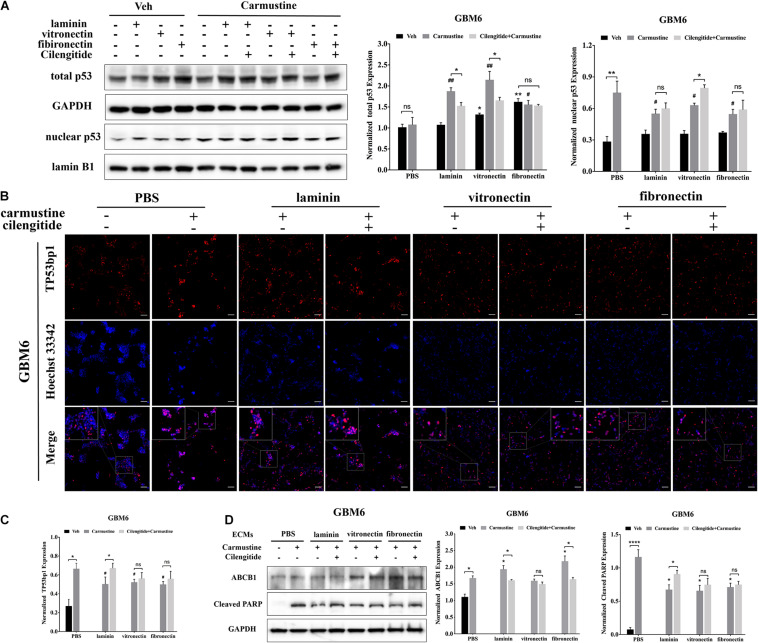
Extracellular matrix (ECM) suppresses p53-mediated apoptosis and increases ABCB1 expression. **(A)** Representative western blot of GBM6 with or without carmustine and cilengitide [100 μM, phosphate-buffered saline (PBS) as control] treatment on total p53 and nuclear p53 expression (*n* = 3). **(B,C)** Immunofluorescent staining assay of Tp53bp1 expression in GBM6 cells on precoated ECMs. Scale bars, 100 μm. **(D)** Western blot assay of GBM6 with or without carmustine and cilengitide (100 μM, PBS as control) treatment on ABCB1 and cleaved PARP expression (*n* = 3, *p < 0.05, ****p < 0.0001, ^#^*p* <0.05, ^##^*p* < 0.01).

Translocation of p53 to the nucleus, where it acts as a TF to regulate gene expression, is associated with apoptosis in response to DNA damage (Castrogiovanni et al., 2018). Western blots were performed to characterize levels of p53 in the nucleus. In untreated GBM6 cells, total p53 expression was elevated for cells when grown on vitronectin or fibronectin (Figure 4A). However, nuclear expression of p53 was unaffected by the ECM substrate in cells grown on any ECM protein. Expression of nuclear p53 was significantly higher with carmustine treatment in cells all conditions (*p* < 0.05). However, carmustine treatment induced higher expression of total p53 only in cells cultured on laminin and vitronectin. Dual treatment with carmustine and cilengitide significantly reduced total p53 levels, compared with carmustine alone, in GBM6 cells cultured on precoated laminin or vitronectin, but not fibronectin. Nuclear expression of p53 increased in cells on vitronectin substrates with dual treatment, compared with carmustine alone. Dual treatment had no effects on nuclear p53 levels on laminin- or fibronectin-coated substrates. Consistent with TF reporter and Western blot results, quantification of numbers of cells with nuclear TP53bp1 from immunofluorescence images showed elevated nuclear p53 with carmustine treatment and decreased by precoated laminin, vitronectin, and fibronectin, consistent with the nuclear p53 results (Figures 4B,C).

Western blots for c-PARP expression, indicative of apoptotic cells, showed an increased in c-PARP with carmustine treatment in all conditions for GBM6 (Figure 4D), and U87 and HK308 cells (Supplementary Figure S3). However, culture on ECM-coated substrates significantly reduced c-PARP, which was significantly compared with cells on uncoated substrates. In GBM6 cells cultured on laminin, dual treatment with carmustine and cilengitide significantly increased c-PARP levels.

Expression of efflux transporter ABCB1, a product of the MDR1 gene, increased with carmustine treatment in all cases (Figure 4D and Supplementary Figure S3). For carmustine-treated GBM6 cells cultured on precoated laminin or fibronectin, but not vitronectin, substrates, ABCB1 expression was significantly higher than cells on uncoated substrates. Addition of cilengitide to carmustine reduced this increase in ABCB1 expression. Similar patterns of expression for cleaved PARP and ABCB1 were detected in U87MG and HK308 (Supplementary Figure S3).

### Integrin α_*v*_-Mediated FAK/Paxillin/AKT Signaling Pathway Is Essential for Glioblastoma Cell Proliferation

**FIGURE 5:**
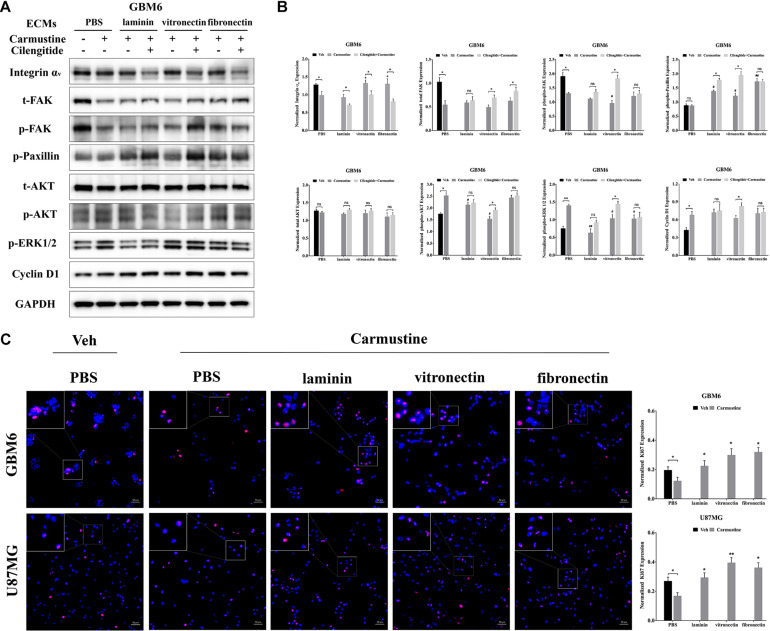
Integrin α_*v*_-mediated FAK/paxillin/AKT signaling pathway is essential for glioblastoma (GBM) cell proliferation. **(A,B)** Western blot assay of GBM6 cells with or without carmustine and cilengitide [100 μM, phosphate-buffered saline (PBS) as control] treatment on integrin α_*v*_, total/p-FAK, paxillin, total/p-AKT, p-ERK 1/2, and cyclin D1 (*n* = 3, *p < 0.05, **p < 0.01, ^#^*p* < 0.05, ^##^*p* < 0.01). **(C)** Immunofluorescent staining assay of Ki67 expression in GBM6 and U87MG cells on precoated extracellular matrices (ECMs) Ki67 positive cells were normalized to total cell numbers. Scale bars, 50 μm (*n* = 3, *p < 0.05, **p < 0.01).

The FAK/paxillin signaling pathway, downstream of integrin α_*v*_, is involved in tumor progression, angiogenesis, and metastasis (Eke and Cordes, 2015; Noh et al., 2020). In the current study, Western blots showed decreased expression of integrin α_*v*_ by GBM6 cells cultured on all substrates after 48 h of treatment with carmustine, when compared with non-treated cells (Figures 5A,B). Integrin α_*v*_ expression was significantly elevated by carmustine-treated GBM6 cells cultured on vitronectin or fibronectin, compared with uncoated or laminin-coated substrates. On all substrates, combined treatment with carmustine and cilengitide significantly reduced expression of integrin α_*v*_ compared with carmustine treatment alone (Figure 5 and Supplementary Figure S6). While integrin α_*v*_ expression was significantly elevated by carmustine treatment in HK308 cells cultured on uncoated or laminin-coated substrates, expression decreased on fibronectin and was unchanged on vitronectin (Supplementary Figure S5). For HK308 cells that adhered to laminin, but not fibronectin or vitronectin, dual treatment with carmustine and cilengitide significantly reduced expression of integrin α_*v*_ compared with carmustine treatment alone. Similarly, expression of integrin α_*v*_ decreased with carmustine treatment in U87 cells on uncoated substrates; however, no significant effects were observed in cells on ECM-coated substrates (Supplementary Figure S4). In contrast to patient-derived GBM6 and HK308 cells, combined treatment with carmustine and cilengitide significantly upregulated expression of integrin α_*v*_ in immortalized U87 cells cultured on vitronectin or fibronectin, when compared with carmustine treatment alone.

While integrin α_*v*_ levels in GBM6 cells decreased when cilengitide was added to carmustine treatment, total levels of FAK increased in cultures on vitronectin and fibronectin (Figure 5). Levels of phosphorylated FAK increased significantly in GBM6 cells cultured on vitronectin, but not laminin, fibronectin, or PBS control (Supplementary Figure S6). Similarly, t-/p-FAK was decreased after carmustine treatment in cells growing on precoated PBS and was not found restored in cells growing on precoated laminin, vitronectin, or fibronectin. Phosphorylation of paxillin, a focal adhesion protein recruited to the intracellular domain of ECM-engaged integrins, was upregulated significantly when GBM6 cells on all ECM-coated, but not uncoated, substrates were treated with carmustine (Figure 5). With dual carmustine and cilengitide treatment, p-paxillin was further increased in cells on laminin and vitronectin.

In addition, p-AKT, p-ERK 1/2, and cyclin D1 were increased in a large degree by carmustine without ECM protein presence. Cilengitide induced a significant elevation of p-AKT, p-ERK 1/2, and cyclin D1 in cells growing on vitronectin. U87MG and HK308 showed variant changes but the same activation trend in FAK/paxillin/AKT signaling pathway (Supplementary Figures S4, S5). In GBM6 and U87MG cells, numbers of proliferating Ki67 positive cells were significantly decreased with carmustine treatment on precoated PBS, but this effect was lost when cells were cultured on precoated laminin, vitronectin, and fibronectin (Figure 5C).

### Increased Activity of Epidermal Growth Factor Receptor-Mediated Pathways Correlates With Increased Cell Survival in Integrin α_*v*_ Knockdowns

**FIGURE 6:**
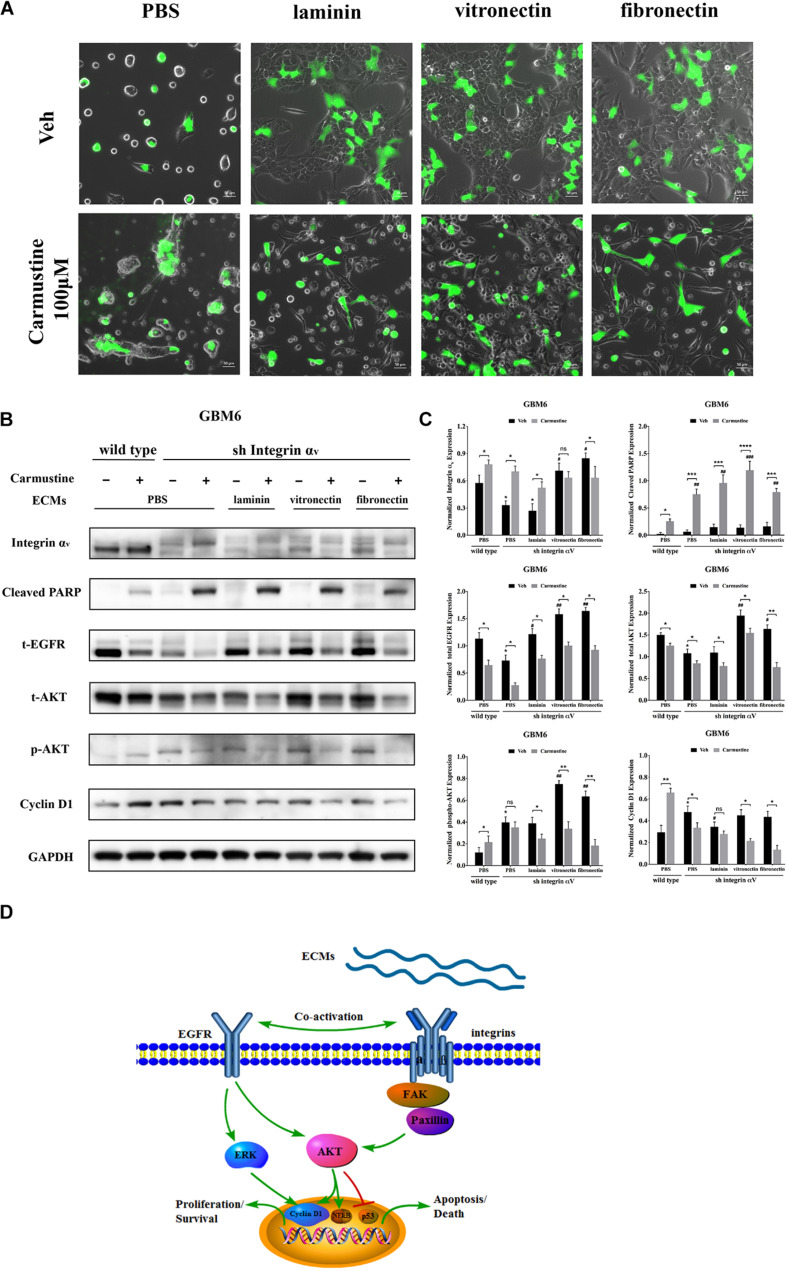
Increased activity of epidermal growth factor receptor (EGFR)-mediated pathways correlates with increased cell survival in integrin α_*v*_ knockdowns. **(A)** Representative light images of GBM6 cells transfected with integrin α_*v*_ shRNA [identified by green carmustine [100 μM, phosphate-buffered saline (PBS) as control] treatment on precoated fibronectin, laminin, and vitronectin. Scale bars, 50 μm. **(B,C)** Western blot assay of wild-type and sh integrin α_*v*_ transfected GBM6 cells with or without carmustine and cilengitide (100 μM, PBS as control) treatment on integrin α_*v*_, c-PARP, total-EGFR, total/p-AKT, and cyclin D1 (*n* = 3, *p < 0.05, **p < 0.01, ***p < 0.001, ****p < 0.0001, ^#^*p* < 0.05, ^##^*p* < 0.01, ^###^*p* < 0.001). **(D)** Hypothetic schematic diagram illustrated the mechanism of extracellular matrices (ECMs) induced cell adhesion-mediated drug resistance (CAMDR) by activation of integrin α_*v*_-mediated FAK/paxillin/AKT signaling pathway and co-activation of EGFR-mediated pathways when integrin α_*v*_ was knocked down.

To investigate the relationship between integrin α_*v*_ and EGFR-mediated oncogenic pathways, integrin α_*v*_ was knocked down in GBM6 cells using a lentiviral vector encoding shRNA against integrin α_*v*_. Green fluorescent protein (GFP) was used to identify transfected cells expressing the shRNA (Figure 6A). Compared with uncoated or laminin-coated substrates, culture on vitronectin- or fibronectin-coated substrates significantly reduced efficacy of the shRNA knockdown of integrin α_*v*_, indicating its importance for cell survival (Figures 6B,C). When treated with carmustine, levels of integrin α_*v*_ in knockdown cultures on uncoated and laminin substrates were elevated to those found in wild-type cultures on uncoated substrates. In contrast, knockdown cultures treated with carmustine on fibronectin further decreased integrin α_*v*_ expression. Strikingly, when integrin α_*v*_ expression was knocked down in GBM6 cells on all substrates, carmustine treatment induced robust apoptosis, with levels of nuclear c-PARP significantly increased over treated, wild-type cells on uncoated substrates.

When integrin α_*v*_ was knocked down, expression of total EGFR increased in GBM6 cells that adhered to laminin, vitronectin, or fibronectin, compared with cells on uncoated substrates (Figures 6B,C). Although total AKT was decreased in integrin α_*v*_ knockdown cells, vitronectin and fibronectin promoted total AKT expression. There was also a significant elevation of phospho-AKT in integrin α_*v*_ knockdown cells growing on precoated vitronectin and fibronectin (Figures 6B,C). Cyclin D1 was decreased remarkably in integrin α_*v*_ knockdown cells treated with carmustine (Figures 6B,C). As shown in Figure 6D, schematic diagram depicted the involvement of integrin α_*v*_ and EGFR for the survival pathway in the CAMDR. Although knockdown of integrin α_*v*_ might prevent the survival of GBM cells, compensatory activation of EGFR and driving its downstream signal molecule AKT and ERK acted as a survival pathway, preventing GBM cells from being killed by carmustine.

## Discussion

Cancer cells may exhibit intrinsic resistance, where they are resistant at the time of initial treatment, and acquired resistance, where resistance develops during the treatment, leading to therapeutic failure. Although a series of publications have revealed the possible mechanisms by which cancer cells become resistant to chemotherapeutic agents, such as decreased loss of receptor or transporter, specific metabolism, enhanced efflux pumps, and limited drug uptake (Zaal and Berkers, 2018), it remains unclear how the tumor microenvironment confers drug resistance to cancer cells. In the current study, our data show three major components of ECM, laminin, vitronectin, and fibronectin, induce GBM cells to become drug resistant by activation of integrin α_*v*_ and EGFR. First, upregulated integrin α_*v*_ activates FAK/paxillin/AKT pathway, leading to a suppression of p53-mediated apoptosis and promoting proliferation by altered cell cycle checkpoints. At the same time, increased expression of ABCB1 indicates that more drug was pumped out by the cells. Second, a compensatory survival pathway was required through activation of EGFR, especially when the integrin α_*v*_ was missing or inhibited by the integrin receptor antagonist cilengitide.

The composition of the ECM in tumors is vastly different from that found in the normal part tissue, but little was known on their exact role in the tumor progression. As the main soluble particles in the ECM, laminin, vitronectin, and fibronectin were found present in the GBM biopsy samples (Schiffer et al., 1984; Shinoda et al., 1989; Gladson and Cheresh, 1991). Similar findings were observed in the current study; by using the TMA, laminin, vitronectin, and fibronectin were expressed in the GBM at a high level. On the contrary, only fibronectin was abundant in the low-grade CNS tumors, indicating that ECM evolved with tumor progression. It was clear from our data that cell attachment was increased when growing on laminin, vitronectin, and fibronectin, enabling GBM cells to acquire the chemotherapeutic resistance, which was also called CAMDR. Our data revealed a variant cell adhesion between cell lines on the same ECM protein, probably as a result of the distribution of certain integrin receptors on the cell surface. Furthermore, integrin receptors have also been shown to bind ECM proteins on sites other than RGD sequences (Albelda and Buck, 1990). Integrins are not the only cell surface receptors for ECM molecules (Venstrom and Reichardt, 1993). CD44 can also mediate glioma cell adhesion to hyaluroonic acid in the ECM and invasion *in vitro* (Xiao et al., 2018). As shown in Figure 1E, fibronectin increased the cell viability in all three cell lines, revealing its strong CAMDR effect, which was also in accordance with the TMA results. It has been reported that fibronectin induced CAMDR in multiple myeloma and bladder cancer (Gao et al., 2017; Wu et al., 2019). Laminins are high-molecular-weight proteins of ECM and are thought as an important and biologically active part of basal lamina, influencing cell differentiation, migration, and adhesion (Yap et al., 2019; Barros et al., 2020). It is also documented that laminin was involved in the acquired chemotherapeutic resistance in gastric cancer (Sun et al., 2014). Only HK308 attached to laminin developed drug resistance in our study.

Cilengitide is based on the cyclin peptide cyclo (-RGDfV-), which is a selective for integrin α_*v*_ and is reported beneficial for GBM patients only in phase II trials (Reardon et al., 2008; Khasraw et al., 2016). Here, cilengitide had different antagonistic effects on GBM6, U87MG, and HK308. As shown in Supplementary Figure S2 and Figure 2, cilengitide detached U87MG completely when cells were not grown on ECM, and more cells detached after vitronectin treatment. A similar result was found in GBM6 in that cells grown on vitronectin more easily detached after carmustine treatment. On the contrary, only attachment to fibronectin was not influenced by cilengitide. Hence, we assume that predominance of integrin receptors of vitronectin is more enriched over other types of receptors on these GBM cells. Expression of vitronectin and its integrin receptor α_*v*_β_3_ have been described in human GBM *in vivo* (Franovic et al., 2015). Other integrin receptors such as integrins β_1_ and β_8_ are also reported in literature (Tchaicha et al., 2011; Cosset et al., 2012). Although integrins β_1_, β_3_ and β_5_ were upregulated in cells grown on vitronectin, there were no changes after the cells were detached by cilengitide. Meanwhile, integrin α_*v*_ was boosted, likely due to the negative feedback response when the cells lost attachment. Since the trend of integrins α and β is not exactly the same, we cannot accurately determine which β subunit plays a major role. Previous literature and research show that fibronectin laminin and vitronectin have different specific integrin receptors. Integrin α_*v*_β_3_, is the main binding receptor of vitronectin (Hermann et al., 1999). Therefore, cilengitide has the most obvious antagonistic effect on cell adhesion to vitronectin compared to on laminin or fibronectin, which is consistent with our current results. In addition to α_*v*_β_3_, α_*v*_β_1_ is also found as a receptor of vitronectin (Ding et al., 2002), whereas the main binding receptors of fibronectin are α_*v*_β_1_ (Ye et al., 2020), α_*v*_β_6_ (Mould et al., 2014), and α_5_β_1_ (Mould et al., 2014) and the main binding receptor of laminin is α_6_β_1_ (Corsini and Martin-Villalba, 2010). Previous research also confirmed that M21 human melanoma cells not only lost the ability to attach to vitronectin but showed a dramatic reduction in tumorigenicity when lacking integrin α_*v*_ gene expression (Lacaria et al., 2020). In addition, accompanied with the elevation of integrin α_*v*_, in our study EGFR was also found upregulated in carmustine- and cilengitide-treated cells growing on vitronectin. Other researchers have proposed that integrin α_*v*_ associates with the EGFR on the cell membrane in a macromolecular complex including the adaptor protein p130Cas and the c-Src kinase that could lead to phosphorylation of specific EGFR tyrosine residues (Kosibaty et al., 2020).

Many studies have addressed the association between NFκB and cell adhesion events. NFκB activity is required for the expression of several cell adhesion molecules such as vascular cell adhesion molecule-1 (VCAM-1), intracellular adhesion molecule-1 (ICAM-1), and endothelial leukocyte adhesion molecule-1 (ELAM-1) (Ampofo et al., 2019). Previous publication also reported that over-expression of NFκB subunits in GBM cells elevated the levels of fibronectin gene expression, indicating a positive loop in the regulatory role for NFκB in ECM protein–cell communication. Activation of NFκB also led to an increase in the levels of mRNA for the α_*v*_ and β_3_ integrin subunits, which was in accordance with our present results (Ritchie et al., 2000). Bioluminescent analysis of genetic reporters has been previously used in live cells to assess dynamic changes in TF activity (Pannier et al., 2007; Penalver Bernabe et al., 2016). Our data indicated that activity of NFκB was promoted by either drug treatment as a feedback response or increased attachment to vitronectin and fibronectin, but detachment induced by cilengitide dramatically suppressed activity of NFκB. The result was in accordance with the prior studies showing that NFκB was involved in the proliferation progress in many tumors (Zeligs et al., 2016). Another interesting thing was that the activity of p53 was only elevated by carmustine in cells growing on precoated PBS but decreased in cells growing on laminin, vitronectin, and fibronectin. Along with the downregulation, p53-mediated apoptosis was reduced accordingly. It has been reported that high expression of fibronectin is associated with the cell proliferation and malignancy via NFκB/p53-apoptosis signaling pathway in colorectal cancer (Yi et al., 2016).

A number of studies have demonstrated that chemotherapeutic resistance against alkylating agents is often associated with the overexpression of ABCB1 (Munoz et al., 2014; Stavrovskaya et al., 2016). Generally, ABCB1 serves as a drug efflux pump actively reducing intracellular drug concentrations in resistant tumor cells, but its biological regulation remains unclear. We found that ABCB1 was elevated by the treatment of carmustine without precoated ECM proteins, which is probably due to negative feedback to the alkylating agent. ABCB1 went to an even higher-level expression with precoated fibronectin, which was correlated with the upregulation of EGFR, as was also reported in the prior work (Munoz et al., 2014). Among microenvironment–cell interaction-mediated regulation of ABCB1, a family of ECM proteins called CCN (CYR61/CTGF/NOV) was demonstrated to regulate ABCB1 and to confer vinblastine resistance in renal cell carcinoma cells targeting α_*v*_ β_3_ (Long et al., 2013). HA–CD44 interactions have been shown to be involved in multidrug resistance in breast tumor cells and are linked to a positive feedback circuit involving HA, phosphoinositide 3-kinase (PI3K), and ErbB2 (Ma et al., 2018).

As shown in the present study, activation of integrin α_*v*_-mediated FAK/paxillin/AKT signaling pathway was required for the proliferation, which was consistent with studies in other tumor cells that integrin α_*v*_ facilitates a proliferative role (Hayashido et al., 2014; Hung et al., 2014). Some research also indicated the integrin α_*v*_ was involved in the GBM neurosphere formation. Integrin α_*v*_-mediated cell–cell adhesion limited cell dispersion from spheroids in a fibronectin-poor microenvironment. However, in a fibronectin-rich microenvironment, α_*v*_ promoted cell dispersion (Blandin et al., 2016). EGFR has been well identified as a predictor for the chemoresistance of GBM in the last decade (Taylor et al., 2012). Here, we show that with integrin α_*v*_ knock down, compensatory activation of EGFR may enable the cells to escape apoptotic effects of alkylating agents. Overall, this study emphasizes that importance of interactions of the ECM and a corresponding profile of cell surface receptors to regulation of cancer cell survival.

## Data Availability Statement

The original contributions presented in the study are included in the article/Supplementary Material, further inquiries can be directed to the corresponding author/s.

## Ethics Statement

The patient-derived GBM cell lines were in accordance with UCLA Institutional Review Board protocol 10-000655.

## Author Contributions

SS and QY conceived the studies and wrote the manuscript. QY and WX designed the methodology. QY, WX, SS, AS, and JL conducted the experiments and acquired and analyzed the data. All authors contributed to the article and approved the submitted version.

## Conflict of Interest

The authors declare that the research was conducted in the absence of any commercial or financial relationships that could be construed as a potential conflict of interest.

## Supplementary Material

Supplementary Figure 1 **(A)** Representative light images of U87MG cells with or without carmustine (100 μM, PBS as control) treatment on precoated fibronectin, laminin, and vitronectin. Scale bars, 50 μm. **(B)** Representative light images of HK308 cells with or without carmustine (100 μM, PBS as control) treatment on precoated fibronectin, laminin, and vitronectin. Scale bars, 50 μm.

Supplementary Figure 2 **(A)** Cell viability assay of carmustine and cilengitide on U87MG and HK308 cells. Error bars, *SD* (*n* = 3, ^∗^*p* < 0.05, ^∗∗^*p* < 0.01, ^∗∗∗^*p* < 0.001, ^#^*p* < 0.05, ^##^*p* < 0.01). **(B)** Representative light images of U87MG cells with or without carmustine and cilengitide (100 μM, PBS as control) treatment on precoated fibronectin, laminin, and vitronectin. Scale bars, 50 μm. **(C)** Representative light images of HK308 cells with or without carmustine and cilengitide (100 μM, PBS as control) treatment on precoated fibronectin, laminin, and vitronectin. Scale bars, 50μm.

Supplementary Figure 3 **(A)** Western blot assay of U87MG cells with or without carmustine and cilengitide (100 μM, PBS as control) treatment on ABCB1 and Cleaved PARP expression (*n* = 3, ^∗^*p* < 0.05, ^∗∗^*p* < 0.01, ^∗∗∗^*p* < 0.001, ^#^*p* < 0.05, ^###^*p* < 0.001). **(B)** Western blot assay of HK308 cells with or without carmustine and cilengitide (100μM, PBS as control) treatment on ABCB1 and Cleaved PARP expression (*n* = 3, ^∗^*p* < 0.05, ^∗∗∗^*p* < 0.001, ^#^*p* < 0.05, ^###^*p* < 0.001).

Supplementary Figure 4 **(A,B)** Western blot assay of U87MG cells with or without carmustine and cilengitide (100 μM, PBS as control) treatment on integrin α_*v*_, total/p-FAK, paxillin, total/p-AKT, p-ERK 1/2, and cyclin D1 (*n* = 3, ^∗^*p* < 0.05, ^∗∗^*p* < 0.01, ^∗∗∗^*p* < 0.001, ^#^*p* < 0.05).

Supplementary Figure 5 **(A,B)** Western blot assay of HK308 cells with or without carmustine and cilengitide (100 μM, PBS as control) treatment on integrin α_*v*_, total/p-FAK, paxillin, total/p-AKT, p-ERK 1/2, and cyclin D1 (*n* = 3, ^∗^*p* < 0.05, ^∗∗^*p* < 0.01, ^#^*p* < 0.05).

Supplementary Figure 6 **(A,B)** Western blot assay of GBM6 cells with dual treatment of carmustine and cilengitide on uncoated ECMs (100 μM, PBS as control) (*n* = 3, ^∗^*p* < 0.05, ^∗∗^*p* < 0.01, ^∗∗∗^*p* < 0.001, ^#^*p* < 0.05, ^##^<0.01, ^###^<0.001).
