# Supplementary material for: Extracellular Matrix Proteins Confer Cell Adhesion-Mediated Drug Resistance Through Integrin αv in Glioblastoma Cells
Source: Front Cell Dev Biol. 2021 Mar 23;9:616580. doi: 10.3389/fcell.2021.616580 (PMC8021872; doi:10.3389/fcell.2021.616580)
Supplement: Supplementary file 1 [file Table_1.DOCX]

**Sequences of enhancer regions**

| TF | Sequence Information | Reference | Full Name |
| --- | --- | --- | --- |
| p53 | TACAGAACATGTCTAAGCATGCTGTGCCTTGCCTGGACTTGCCTGGCCTTGCCTTGGG | Panomics | N/A |
| NFκB | GGGAATTTCCGGGAATTTCCGGGAATTTCCGGGAATTTCCGGGAATTTCCGGGAATTTCC | Panomics | nuclear factor binding enhancer element of immunoglobulin kappa light-chain in activated B cells |
| c-myc | GCTAGCCACGTGCACGTGCACGTGCACGTGCACGTGACCGGT | Transfac ID R02207 | N/A |

TF: transcriptional factor
