# Supplementary figures and images for: Extracellular Matrix Proteins Confer Cell Adhesion-Mediated Drug Resistance Through Integrin αv in Glioblastoma Cells
Source: Front Cell Dev Biol. 2021 Mar 23;9:616580. doi: 10.3389/fcell.2021.616580 (PMC8021872; doi:10.3389/fcell.2021.616580)

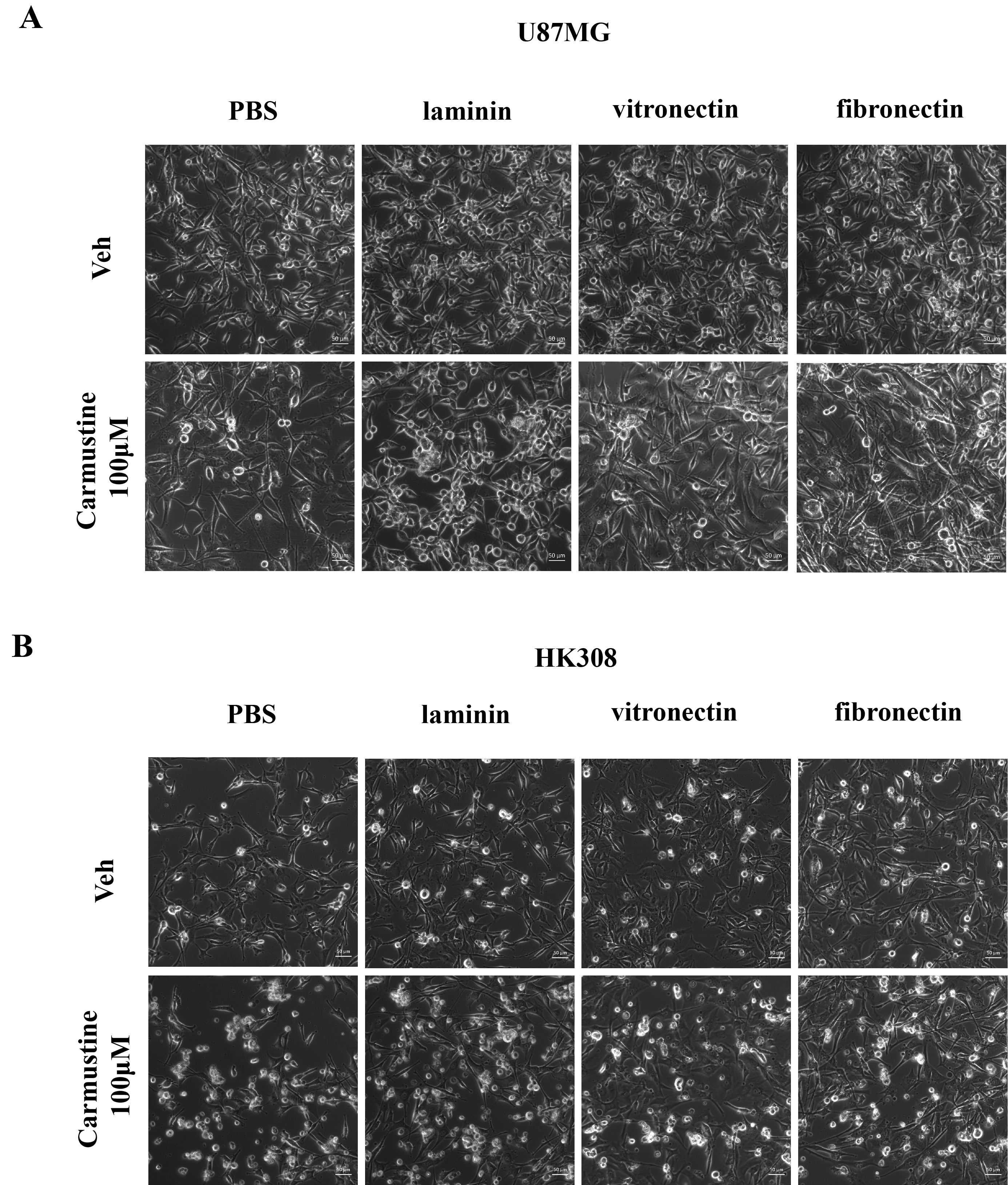

Supplement: Supplementary Figure 1 — (A) Representative light images of U87MG cells with or without carmustine (100 μM, PBS as control) treatment on precoated fibronectin, laminin, and vitronectin. Scale bars, 50 μm. (B) Representative light images of HK308 cells with or without carmustine (100 μM, PBS as control) treatment on precoated fibronectin, laminin, and vitronectin. Scale bars, 50 μm. [file Image_1.JPEG]

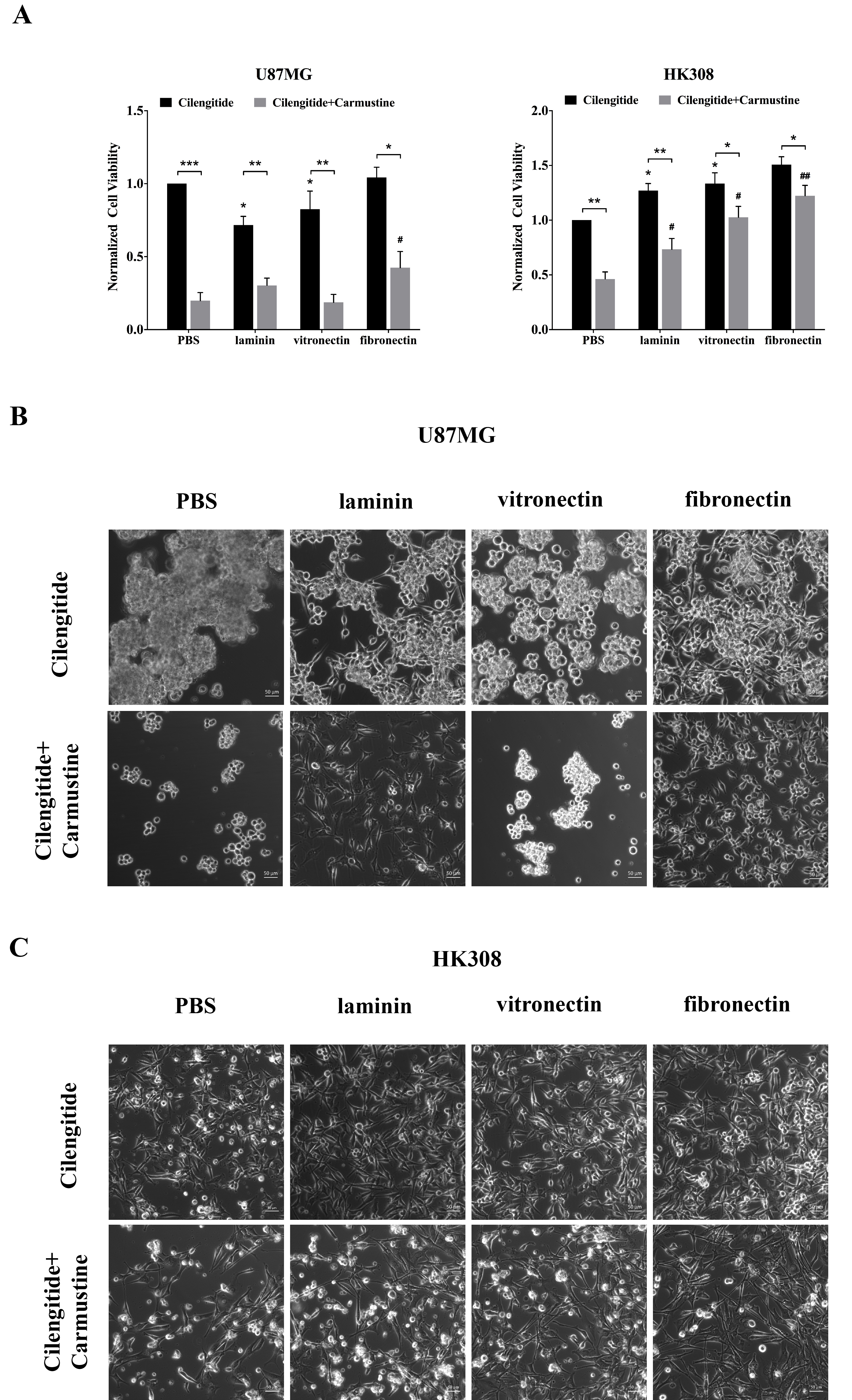

Supplement: Supplementary Figure 2 — (A) Cell viability assay of carmustine and cilengitide on U87MG and HK308 cells. Error bars, SD (n = 3, ∗p < 0.05, ∗∗p < 0.01, ∗∗∗p < 0.001, #p < 0.05, ##p < 0.01). (B) Representative light images of U87MG cells with or without carmustine and cilengitide (100 μM, PBS as control) treatment on precoated fibronectin, laminin, and vitronectin. Scale bars, 50 μm. (C) Representative light images of HK308 cells with or without carmustine and cilengitide (100 μM, PBS as control) treatment on precoated fibronectin, laminin, and vitronectin. Scale bars, 50μm. [file Image_2.JPEG]

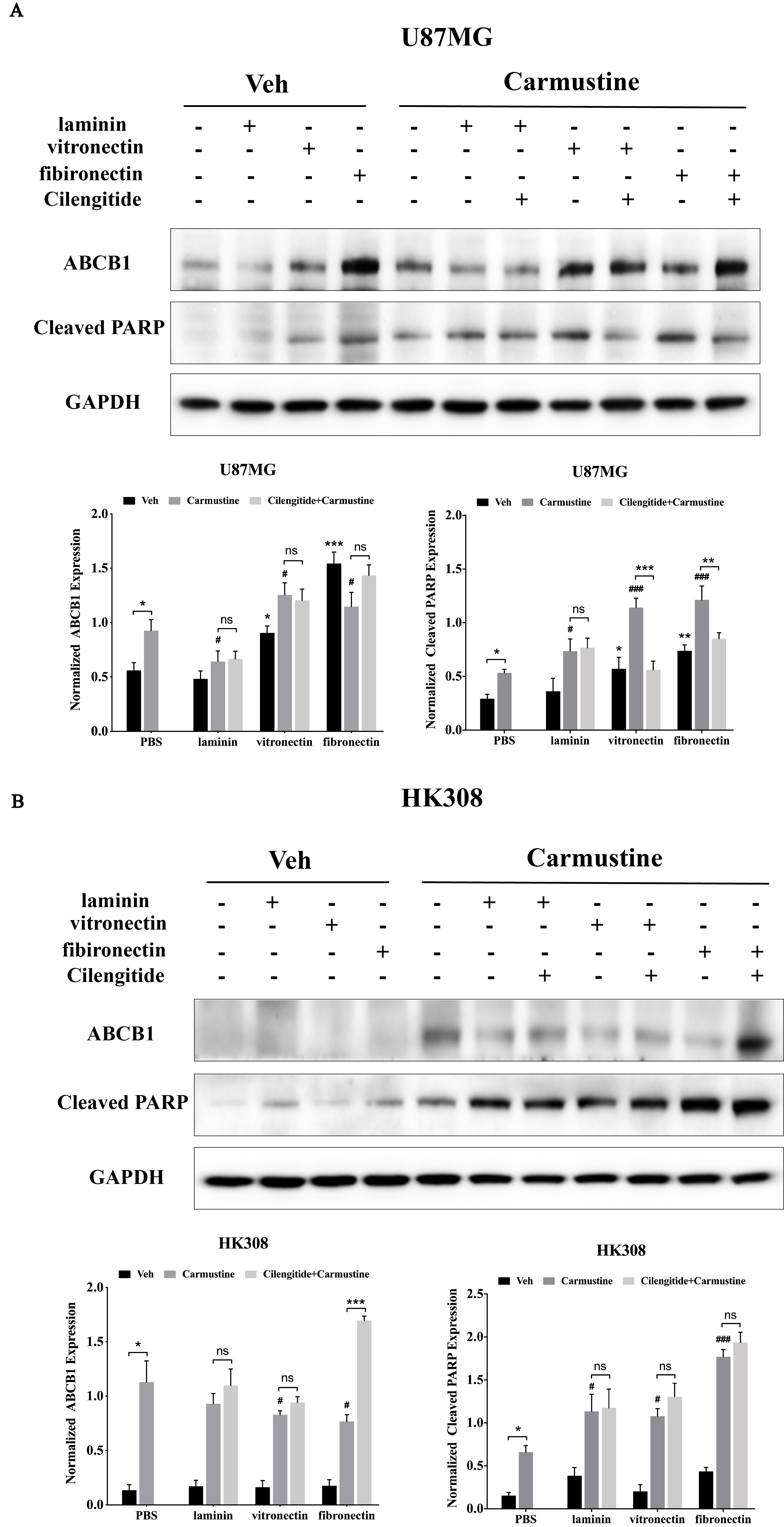

Supplement: Supplementary Figure 3 — (A) Western blot assay of U87MG cells with or without carmustine and cilengitide (100 μM, PBS as control) treatment on ABCB1 and Cleaved PARP expression (n = 3, ∗p < 0.05, ∗∗p < 0.01, ∗∗∗p < 0.001, #p < 0.05, ###p < 0.001). (B) Western blot assay of HK308 cells with or without carmustine and cilengitide (100μM, PBS as control) treatment on ABCB1 and Cleaved PARP expression (n = 3, ∗p < 0.05, ∗∗∗p < 0.001, #p < 0.05, ###p < 0.001). [file Image_3.JPEG]

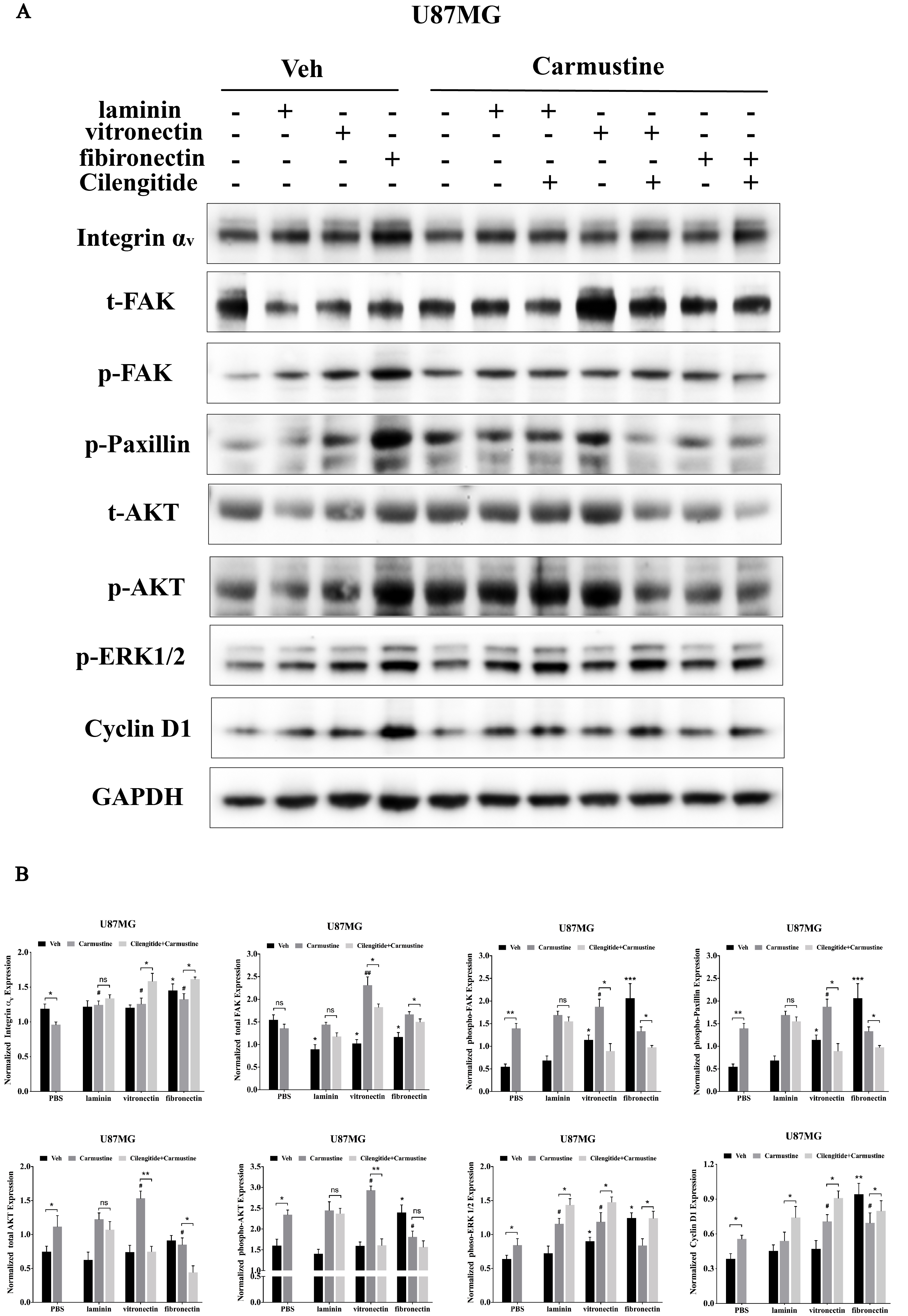

Supplement: Supplementary Figure 4 — (A,B) Western blot assay of U87MG cells with or without carmustine and cilengitide (100 μM, PBS as control) treatment on integrin αv, total/p-FAK, paxillin, total/p-AKT, p-ERK 1/2, and cyclin D1 (n = 3, ∗p < 0.05, ∗∗p < 0.01, ∗∗∗p < 0.001, #p < 0.05). [file Image_4.JPEG]

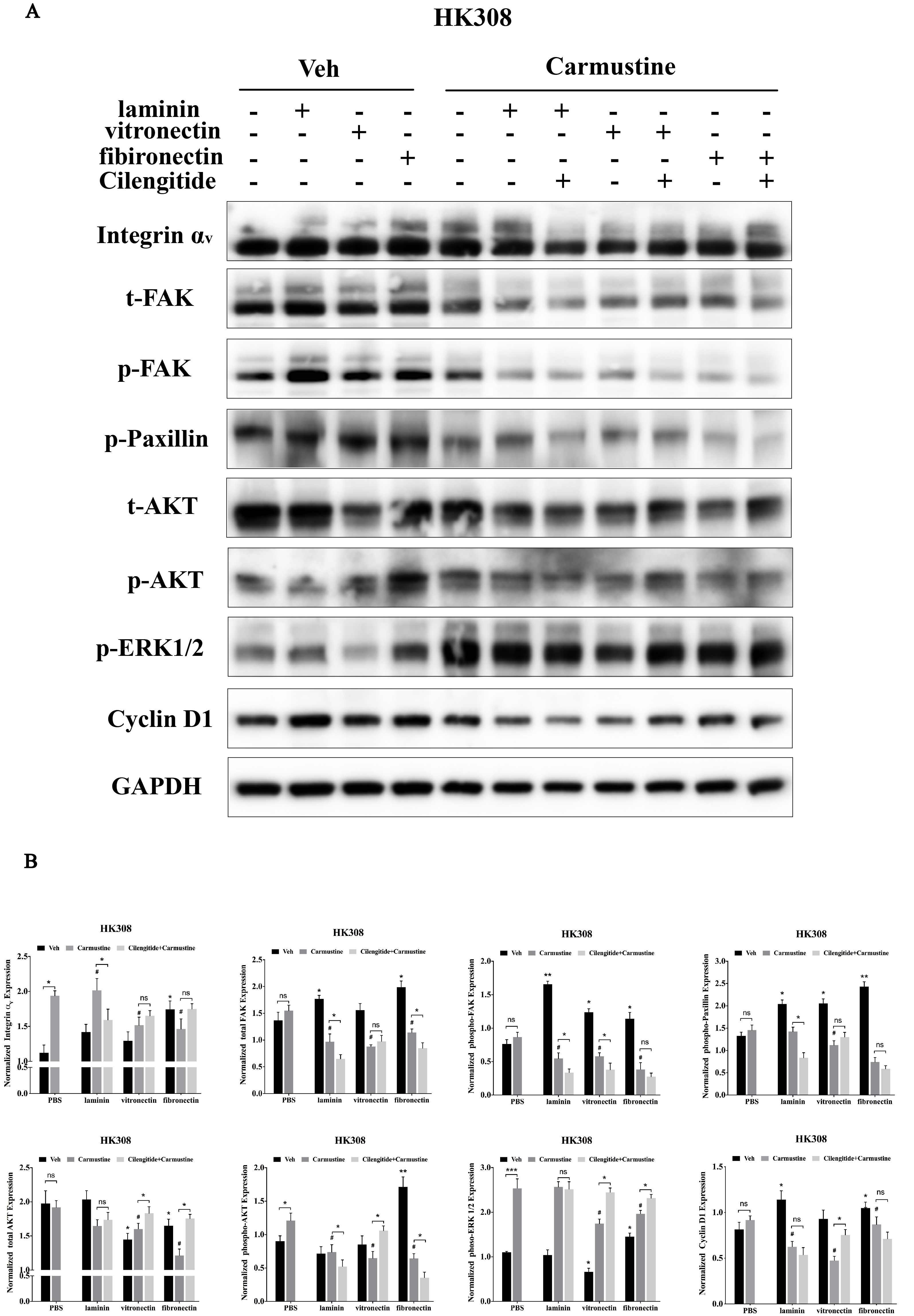

Supplement: Supplementary Figure 5 — (A,B) Western blot assay of HK308 cells with or without carmustine and cilengitide (100 μM, PBS as control) treatment on integrin αv, total/p-FAK, paxillin, total/p-AKT, p-ERK 1/2, and cyclin D1 (n = 3, ∗p < 0.05, ∗∗p < 0.01, #p < 0.05). [file Image_5.JPEG]

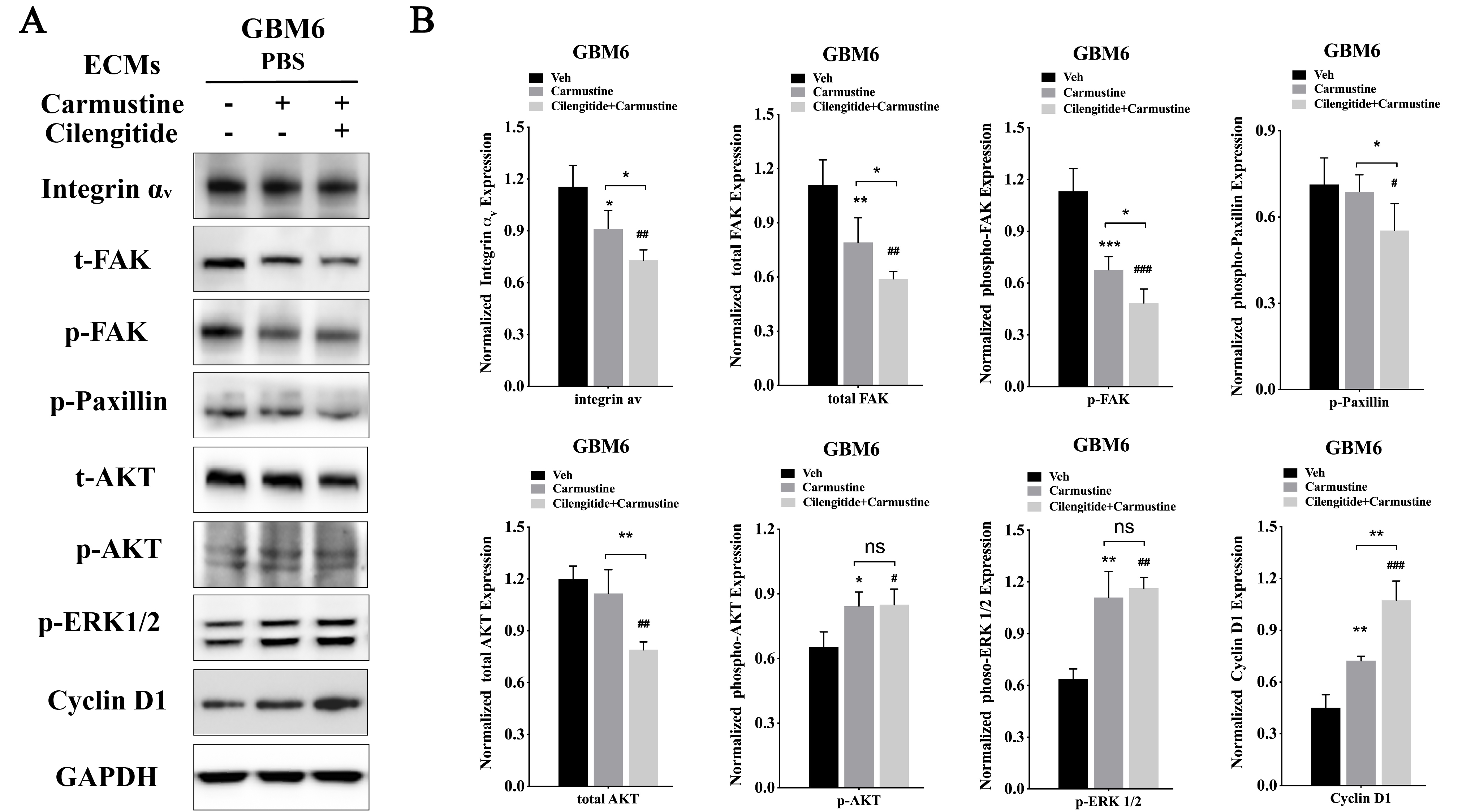

Supplement: Supplementary Figure 6 — (A,B) Western blot assay of GBM6 cells with dual treatment of carmustine and cilengitide on uncoated ECMs (100 μM, PBS as control) (n = 3, ∗p < 0.05, ∗∗p < 0.01, ∗∗∗p < 0.001, #p < 0.05, ##<0.01, ###<0.001). [file Image_6.JPEG]
